# HDL in Endocrine Carcinomas: Biomarker, Drug Carrier, and Potential Therapeutic

**DOI:** 10.3389/fendo.2018.00715

**Published:** 2018-11-30

**Authors:** Emily E. Morin, Xiang-An Li, Anna Schwendeman

**Affiliations:** ^1^Department of Pharmaceutical Sciences, College of Pharmacy, University of Michigan, Ann Arbor, MI, United States; ^2^BioInterfaces Institute, University of Michigan, Ann Arbor, MI, United States; ^3^Department of Physiology, Saha Cardiovascular Research Center, College of Medicine, University of Kentucky, Lexington, KY, United States

**Keywords:** High-density lipoprotein (HDL), Apolipoprotein A-I (ApoA-I), endocrine cancer, cholesterol, cancer therapy

## Abstract

High-density lipoprotein (HDL) have long been studied for their protective role against cardiovascular diseases, however recently relationship between HDL and cancer came into focus. Several epidemiological studies have shown an inverse correlation between HDL-cholesterol (HDL-C) and cancer risk, and some have even implied that HDL-C can be used as a predictive measure for survival prognosis in for specific sub-population of certain types of cancer. HDL itself is an endogenous nanoparticle capable of removing excess cholesterol from the periphery and returning it to the liver for excretion. One of the main receptors for HDL, scavenger receptor type B-I (SR-BI), is highly upregulated in endocrine cancers, notably due to the high demand for cholesterol by cancer cells. Thus, the potential to exploit administration of cholesterol-free reconstituted or synthetic HDL (sHDL) to deplete cholesterol in endocrine cancer cell and stunt their growth of use chemotherapeutic drug loaded sHDL to target payload delivery to cancer cell has become increasingly attractive. This review focuses on the role of HDL and HDL-C in cancer and application of sHDLs as endocrine cancer therapeutics.

## Introduction

Endocrine cancers are defined as those affecting the hormone secreting tissues of our body, including cancers of the adrenal, thyroid, parathyroid, prostate, pancreatic, and reproductive tissues. A rare subset of endocrine cancers, called neuroendocrine tumors (NETs), are neoplasms originating in endocrine tissue that migrate to form hormone-secreting tumors in other organs of the body, including intestine, lung, and pancreas (1). While specific molecular signatures may vary among the different types of endocrine cancers, they all share a common modality which is essential for tumor cell proliferation and overall survival: a high demand for cholesterol (2–4).

Cholesterol is a precursor molecule for steroid synthesis and bile acid production, making it essential for hormone production by endocrine tissue (5). Cholesterol is also an important component of cellular membranes, offering structure and rigidity to the plasma membrane as well as clustering with sphingolipids and glycerophospholipids to form highly-stable membrane microdomains or “lipid rafts” that host a number of proteins and lipids involved in key cell signaling pathways (6). In cancer, rapid cellular division is needed for the growth and survival of the tumor. Hence, a large demand for cholesterol is needed to facilitate the rapid formation of new membranes (3). Endocrine cancers in particular, display an even higher demand for cholesterol due to increased hormone and steroid production by these cells (7, 8).

A hydrophobic molecule, cholesterol has very poor aqueous solubility and thus is transported throughout the body by lipoproteins. Under normal conditions, HDL is a key participant in the reverse cholesterol transport (RCT) pathway, a process by which excess cholesterol from peripheral tissue is taken up by HDL and transported back to the liver for secretion in the bile or for redistribution to endocrine tissue for steroid production. Historically, HDL-C has been the focus of lipid metabolism modulating therapeutics for cardiovascular diseases, as high HDL-C or “the good cholesterol” and low LDL-C “bad cholesterol” have been well established as markers of cardiovascular health. Specifically, nascent HDL has the ability to reduce the burden of atherosclerosis by depleting foam-cell macrophages of their cholesterol and reducing inflammation and oxidation in the surrounding atheroma environment (9, 10). In this review, we will summarize what is known about the association between HDL-C levels and cancer and examine the utility of reconstituted or synthetic HDL as a potential therapeutic and drug delivery vehicle for endocrine cancers.

## HDL-C in cancer

HDL is an endogenous, nanosized particle composed apolipoproteins, and lipids (11). Naturally, these particles range in shape, size, density, and charge depending on their lipid composition, protein cargo, and degree of maturation (11). The main protein component of HDL, apolipoprotein A-I (ApoA-I), is initially synthesized in the liver where it is subsequently secreted into the circulation. Once secreted, ApoA-I picks up a small amount of lipid to form pre-β HDL particles. These nascent, cholesterol-poor discs can then further interact with cholesterol-rich cells of the periphery to take up and deliver that cholesterol back to the liver where it is taken up via scavenger receptor type B-I (SR-BI) for secretion or further processing. Once picked up by HDL, cholesterol is esterified by lecithin:acyl cholesterol transferase (LCAT) to form cholesterol ester. Cholesterol ester is then buried within HDL's hydrophobic lipid core, inducing the maturation and formation of larger, spherical HDL particles. Spherical HDL particles contain not only ApoA-I, but also ApoE, which facilitates the growing load of CE into the hydrophobic core since ApoA-I can only facilitate a limited amount of CE in the HDL core. ApoE is also useful in that it is a substrate for low-density lipoprotein receptor (LDLR) and can deliver HDL cargo to hepatic LDLR for biliary excretion or to endocrine tissue expressing LDLR or SR-BI for use in steroid production (12, 13).

HDL is highly heterogeneous and is present in a variety of different forms depending on its size, shape, density, and lipid/protein composition. This is a result of HDL remodeling, which is a continuous process involving several endogenous enzymes (14). Put simply, HDL can be continually and reversibly recycled between lipid-poor apoA1, discoidal HDL, and small/large/larger spherical HDLs. These subsets of HDL are classified into two groups, HDL_2_ and HDL_3_, based on their densities (15, 11). HDL_2_ is lipid-rich and less dense (1.063–1.125 g/mL) than its HDL_3_ counterpart, which is dense (1.125–1.21 g/mL) protein-rich in comparison (11). Both HDL_2_ and HDL_3_ can be further divided into 2 and 3 subclasses, respectively, based on their size; HDL_3_ ranges in size from roughly 7–9 nm in diameter while HDL_2_ ranges from about 9–12 nm (11). To further complicate things, HDL can also be classified according to its surface charge and shape. Spherical, more neutral HDL particles are classified as α-HDL, while nascent, discoidal HDL particles, known as β-HDL, are poorly lipidated and more negative in overall charge.

In addition to the existing variety of subpopulations in healthy individuals, HDL particle makeup can vary significantly among patients of different disease states (16). Particularly, recent studies have identified changes in the diverse proteome of HDL particles in the various disease states (17, 18). While ApoA-I is the main protein in HDL, other proteins including ApoA-II, ApoC, paraoxanase (PON), ApoM, and serum amyloid A (SAA) have been identified and can be altered under disease conditions (19–21). The lipid composition of HDL particles can also vary with disease (22), and chronic changes in the HDL lipidome have been attributed to the high inflammatory state of various diseases, including the presence of lysophosphatidic acid (LPA), a phospholipid implicated in the progression of several endocrine cancers (23–25). Under such conditions, including atherosclerosis and lupus, HDL isolated from patients is said to be dysfunctional or proinflammatory, and its abilities to carry out cholesterol efflux and exert anti-inflammatory properties are lost (26–31). Similarly, studies have shown that HDL can promote breast cancer metastasis, which is attributed to the alterations in HDL's lipid and protein compositions under inflammatory and oxidative conditions (32, 33).

### Epidemiology

A number of observational studies and retrospective study analyses have shown that plasma HDL-C and ApoA-I levels are significantly reduced in cancer patients, including those with breast, ovarian, colon, prostate, and pancreatic carcinomas (34–50). These studies are summarized in Table 1. A number of studies also sought to investigate the predictive power of HDL-C or ApoA-I levels in subsets of cancers and found that, when combined with other traditional cancer biomarkers cancer antigen 125 (CA125) and transthyretin (TTR), either ApoA-I or HDL-C levels significantly increased the power of these panels to predict patient prognosis (52, 54–58). In some cases, however, there were no significant associations between HDL-C, ApoA-I, and cancer risk (51). This is likely due to differences in study design and evaluation as well as the methods used to quantify HDL-C and ApoA-I. For example, direct measurements of HDL-C are generally performed by mass precipitation and can be confounded by the presence of ApoE and other proteins. Other methods directly measure HDL particles via size and charge separation using density gradient ultracentrifugation, gel filtration, high performance liquid chromatography (HPLC), and nuclear magnetic resonance (NMR) among others. Each of these techniques has its drawbacks, and is generally bias toward one or more subpopulation of HDL or risks chemical modification of the particles during sample preparation (61). In addition other confounding factors such as lifestyle factors, co-morbidities, and physiological factors (i.e., pre- vs. post-menopausal women), all contributed to heterogeneity of the results since most of the analyses were done retrospectively using the existing body of publicly available clinical trial data. On the other hand, there are studies describing positive correlations between HDL-C and cancer risk, namely in breast cancers (60). However, given the high heterogeneity in HDL proteome, lipidome, and subclass distribution between patients in different disease settings, it is reasonable that such variability exists between studies. While the verdict is still out on the utility of HDL-C and ApoA-I as predictive biomarkers in cancers, there is clearly a role for HDL in this complex disease which will be discussed in more detail to follow.

**Table 1:** Clinical relationships between HDL-C and endocrine cancers.

**Cancer**	**Study design**	**Major findings**	**References**
Breast	• Preoperative serum lipid profile (TC, TG, HDL-C, LDL-C, ApoAI, ApoB) and the clinical data were retrospectively collected for 1,044 breast cancer patients undergoing operation • Kaplan-Meier method and the Cox proportional hazards regression model were used in analyzing the OS and DFS	• Preoperative lower TG and HDL-C level were risk factors of breast cancer patients • Decreased HDL-C associated with lower OS rate • Decreased TG associated with lower DFS rate	(49)
Multiple	• Twenty-six studies including 24,655 individuals identified via PubMed and EMBASE • Meta-analysis to investigate the prognostic significance of serum blood TC, TG, HDL-C, and LDL-C for cancer	• Patients with higher HDL-C had a 37% reduced risk of death compared with lower HDL-C • DFS patients with higher HDL-C level had the risk of disease relapse reduced by 35% compared with patients with lower levels.	(50)
Breast	• Examined the possible association of low HDL-C with incidence of breast cancer using data from the Atherosclerosis Risk in Communities Study (ARIC) cohort • Among 7,575 female members of the ARIC cohort, 359 cases of incident breast cancer were ascertained during the follow-up from 1987 through 2000	• No association of low baseline HDL-cholesterol (< 50 mg/dL) with incident breast cancer in the total sample and a modest association among women who were pre- menopausal at baseline. No association was observed among women who were post-menopausal at baseline • Low HDL-cholesterol among pre-menopausal women may be a marker of increased breast cancer risk	(51)
Multiple	• Assess the relationships of TC, TG, HDL-C, ApoA, ApoB-100, Lp(a) with risk of common cancer forms, and total cancer mortality in comparison to incidence and mortality of CVD • Case-cohort sample out of the prospective EPIC–Heidelberg study, including a random subcohort (*n* = 2,739), and cases of cancer (*n* = 1,632), cancer mortality (*n* = 761), CVD (*n* = 1,070), and CVD mortality (*n* = 381).	• High TC, HDL-C, ApoA, and Lp(a) levels were associated with a reduction in total cancer mortality • High levels of apoB-100 and TG were inversely associated, and high HDL-C levels were positively associated with breast cancer risk • Higher levels of Lp(a) were associated with an increase in prostate cancer risk	(48)
Multiple	• Serum TC, LDL-C, HDL-C, and TG were analyzed in 530 patients with newly diagnosed cancer (97 with hematological malignancies, 92 with tumor of the lung, 108 of the upper diges- tive system, 103 of colon, 32 of breast, and 98 of the genitourinary system) and in 415 non-cancer subjects	• TC, LDL-C, HDL-C, SA, and BMI were significantly lower in cancer than in non-cancer subjects; similar trend for metastatic vs. non-metastatic cancer patients • Lowest values of TC, LDL-C, and HDL-C recorded in patients with hematological malignancies • Highest values of TC, LDL-C, and HDL-C in patients with breast tumor	(36)
Renal cell carcinoma	• Preoperative serum lipid-profile (TC, TG, HDL-C, LDL-C, ApoA- I, and ApoB) were retrospectively performed in 786 patients with RCC	• Patients with low ApoA-I (< 1.04) had significantly lower OS than the high ApoA-I • In the 755 patients with nonmetastasis, the low ApoA-I group was also associated with shortened DFS time compared to the high ApoA-I group	(47)
Pancreatic	• Identify and validate new biomarkers in PCa patient serum samples • 96 serum samples from patients undergoing PCa surgery was compared with sera from 96 healthy volunteers as controls.	• Apolipoprotein A-II, transthyretin, and apolipoprotein A-I were identified as markers • These identified proteins were decreased at least 2-fold in PCa serum compared with the control group.	(52)
Multiple	• A retrospective cohort study of 14,169 men and 23,176 women with type 2 diabetes to investigate the relationship between HDL cholesterol (HDL-C) and cancer risk among type 2 diabetic patients • During a mean follow-up period of 6.4 years, 3,711 type 2 diabetic patients had a cancer diagnosis	• A significant inverse association between HDL-C and the risk of cancer was found among men and women • Suggests an inverse association of HDL-C with cancer risk among men and women with type 2 diabetes, whereas the effect of HDL-C was partially mediated by reverse causation • Each 15 mg/dL increase in baseline HDL-C was associated with an 8–10% decreased risk of cancer in men and a 1–7% decreased risk of cancer in women with type 2 diabetes	(44)
Breast	• Review and meta-analysis of prospective studies investigating associations between TC, HDL-C, and LDL-C levels and the risk of breast cancer	• Evidence of a modest inverse association between TC and more specifically HDL-C and the risk of breast cancer • No association observed between LDL-C and the risk of breast cancer	(45)
Multiple	• Evaluated the prospective association of total, breast, colorectal, and lung cancers and cancer mortality with lipid biomarkers in 15,602 female health professionals in the Women's Health Study (aged ≥45 y, free of cardiovascular disease and cancer, and without hormone replacement therapy or lipid-lowering medications at baseline) • Included 2,163 incident cancer cases (864 breast, 198 colorectal, and 190 lung cancers) and 647 cancer deaths	• Total cancer risk significantly lower for the highest quartile of ApoA-1 • Significant associations included colorectal and lung cancer risk with HDL cholesterol • LDL cholesterol was not significantly associated with risk of total cancer or any site-specific cancers	(46)
Multiple	• Prospective examination of the association between TC and cancer incidence among 1,189,719 Korean adults enrolled in the National Health Insurance Corporation • Over follow-up, 53,944 men and 24,475 women were diagnosed with a primary cancer	• High TC (≥240 mg/dL) was positively associated with prostate cancer and colon cancer in men and breast cancer in women • Higher TC was associated with a lower incidence of liver cancer, stomach cancer, and, in men, lung cancer • TC was inversely associated with all-cancer incidence in both men and women • TC was associated with the risk of several different cancers, although these relationships differed markedly by cancer site	(42)
Multiple	• Examined the relationship between serum HDL-C and risk of overall and site-specific cancers among 29,093 Finnish male smokers in the Alpha-Tocopherol Beta-Carotene (ATBC) study cohort • 7,545 incident cancers were identified during up to 18 years of follow-up	• Higher serum TC inversely associated with cancer risk • Greater HDL-C levels associated with decreased risk of cancer of the lung, prostate, liver, and hematopoietic system • Largely explained by reverse causation	(40)
Multiple	• Systematic analysis of 24 lipid intervention randomized controlled trials (76,265 intervention patients and 69,478 control patients) • Examined association between baseline and on-treatment HDL-C levels and cancer risk	• Significant inverse association between HDL-C and cancer risk • For every 10 mg/dL increase in HDL-C, 28–36% lower risk of developing cancer	(41)
Ovarian, breast, prostate, colon	• A five-center case-control study, involving a retrospective sample of 645 serum specimen • Serum proteomic expressions were analyzed on 153 patients with invasive epithelial OC, 42 with other OC, 166 with benign pelvic masses, and 142 healthy women • Utilized a ProteinChip Biomarker System and SELDI-TOF-MS	• Three biomarkers identified as biomarkers for OC: ApoA-1 (↓in cancer); TT (↓); and a cleavage fragment of ITIH4 (↑)	(53)
Ovarian	• Serum analysis from 31 healthy individuals and 43 from patients with ovarian tumors • Use of micro-LC-MS/MS followed by Western/ELISA to identify five serum protein biomarkers previously reported using SELDI-TOF-MS (54)	• TT (↓), beta-hemoglobin (↑), ApoA-1 (↓), and transferrin (↓)in early-stage OC • When combined with CA125, biomarkers should significantly improve the detection of early stage ovarian cancer	(55)
Ovarian	• Evaluated markers identified by Zhang et al. (53) in an independent study population • Sera from 42 women with OC, 65 with benign tumors, and 76 with digestive diseases • Measured levels of various posttranslationally forms of TTR, apolipoprotein A1, and CA125 using SELDI-TOF-MS • Examined power of markers to discriminate sera from women with ovarian cancer from sera from women with other diseases	• Confirmed findings by Zhang et al. (53) • ApoA-I and TT levels were lower in disease states compared to controls • Markers used alone improved detection of controls with CA125 levels ≥35 units/mL but lost sensitivity for late-stage cases.	(56)
Ovarian	• Evaluated multiplexed bead-based immunoassay of OC-associated biomarkers (TTR and ApoA-1, together with CA125) using serum of 61 healthy individuals, 84 patients with benign ovarian disease, and 118 patients with OC	• Panel of ApoA-I, TT, and CTAPIII combined with CA125 increased sensitivity for detection of early stage OC • Combination of three markers offered maximum separation between non-cancer and stage I/II or all stages of disease	(57)
Ovarian	• Development of multiplexed bead-based immunoassay for detection of known serum biomarkers of cancer (118 OC, 84 benign ovarian disease, 61 healthy controls)	• Combination of transthyretin, and ApoA-I with CA125 improved sensitivity and specificity of OC diagnosis	(58)
Ovarian, breast	• Measured ApoA-I and GPX3 mRNA levels via qRT-PCR in 121 effusions (101 OC, 20 BC) and 85 solid OC specimens (43 primary carcinomas, 42 metastases)	• APOA1 and GPX3 transcript levels were higher in ovarian carcinoma compared with breast carcinoma effusions • APOA1 and GPX3 mRNA levels can effectively differentiate ovarian from breast cancer	(59)
Breast	• Fasting serum samples analyzed for lipid fatty acid and lipoprotein levels • Malignant breast tissue analyzed for hormone receptor binding • 100 women with breast masses (50 malignant, 50 benign)	• Serum lipid and apolipoprotein components of LDL were increased in fibrocystic disease and early stage cancer but decreased in women with early recurrence • Ratio of serum ApoA-1/ApoB levels at time of biopsy was the best predictor of cancer recurrence	(34)
Breast	• Nested case-control study to examine association between HDL-C and breast cancer risk • Serum lipid profiles from 200 age-matched (100 diagnosed before age 50 and 100 at age 50 or older) case-control BC patients	• No difference in HDL-C between BC and control samples • Pre-menopausal cases had significantly lower HDL-C levels than controls • In pre-menopausal cases, each 1 mg/dL increase in HDL-C is associated with a 4% reduction in risk of BC	(35)
Breast	• Estimated the relative risk of breast cancer associated with HDL-C levels using serum samples of 38 823 Norwegian women aged 17–54 years at time of entry • 708 BC cases identified over median follow up time of 17.2 years	• Low HDL-C, as part of the metabolic syndrome, is associated with increased postmenopausal BC risk	(37)
Breast	• Examined relationship between breast cancer and lipid profiles in Taiwanese women • Lipid profiles in fasting serum of 150 BC patients before treatment and 71 healthy controls	• BC patients had significantly lower HDL-C and apoA- I, lower apoA-I/apoB ratios, and higher VLDL-C levels than controls • Lower ApoA-1 and HDL-C levels associated with higher incidence of BC	(38)
Breast	• Nested case-control study from trial containing 4,690 women with extensive mammographic density • Examined whether serial measures of serum lipids and lipoproteins were associated with risk of BC • Measured lipids in an average of 4.2 blood samples for 279 invasive breast cancer case subjects and 558 matched control subjects	• HDL-C and apoA-I were positively associated with BC risk only when HRT was not used	(60)
Endometrial	• Case-control study nested within the European Prospective Investigation into Cancer and Nutrition (EPIC) • Examined the relation between prediagnostic plasma lipids, lipoproteins, and glucose, metabolic syndrome, and EC risk in 284 women with EC and 546 matched controls	• HDL-C levels were inversely correlated with the risk of developing EC • Metabolic abnormalities and obesity may act synergistically to increase risk of developing EC	(39)
Prostate	• Examined the association between serum lipids and prostate cancer risk • A cohort (*n* = 69,735) of all men aged 35 years or older were selected from the Apolipoprotein MOrtality RISk (AMORIS), • Levels of TG, TC, glucose, LDL-C, HDL-C, ApoB, and ApoA-I were measured at baseline, was database • 2,008 men developed prostate cancer	• ApoA-I and HDL levels were inversely associated with prostate cancer risk • Low HDL and ApoA-I as well as increased lipid ratios are related to increased risk of prostate cancer • No association between ApoB, LDL, and non-HDL with prostate cancer risk	(43)

*ApoA-I, apolipoprotein A-I; ApoA-II, apolipoprotein A-II; ApoB, apolipoprotein B; BC, breast cancer; CA125, cancer antigen 125; CTAPIII, connective tissue activating protein III; CTC, circulating tumor cells; DFS, disease-free survival; DMFS, distant-metastasis-free survival; EC, endometrial cancer; HDL-C, High-density lipoprotein cholesterol; IMRT, intensity-modulated radiation therapy; ITIH4, inter-trypsin inhibitor heavy chain H4; LDL-C, low-density lipoprotein cholesterol; OC, ovarian cancer; OS, overall survival; PCa, pancreatic cancer; SELDI-TOF-MS, surface-enhanced laser desorption/ionization time-of-flight mass spectrometry; TC, total cholesterol; TF, transferrin; TG, triglycerides; TT, truncated transthyretin; TTR, transthyretin; VLDL-C, very low-density lipoprotein cholesterol*.

### Mechanism

Whether decreased levels of HDL-C are a causal or consequential factor to cancer progression is yet to be elucidated, however we are logically drawn to the latter. It is known that cancer cells, in particular prostate, adrenal, and breast cancer cells, highly express the SR-BI on their plasma membrane (62–64). Because of their high-demand for cholesterol, cancer cell upregulation of SR-BI is likely a survival mechanism to increase HDL-C recruitment and, thus, increase cholesterol uptake needed for proliferation and hormone production while consequently decreasing circulating HDL-C. That being said, this argument could also be used to explain why, in some reports, HDL-C is associated with an increased risk of cancer, as it continues to provide additional cholesterol to and fuel the growth of the tumor. Regardless, we can take away several key points from these findings: (i) SR-BI is overexpressed in cancer cells, (ii) HDL-C levels are significantly affected by the presence and development of cancer, and (iii) the high affinity between SR-BI and HDL facilitates the transport of cholesterol to/from HDL and the cancer cell. In addition, HDL is known to have potent antioxidant activity and both endogenous and reconstituted HDL particles were shown to inhibit oxidative-stress induced proliferation of pancreatic cells *in vitro* (65). And although the details of the epidemiology can be disputed, there are clear opportunities for therapeutic intervention by utilizing the HDL/SR-BI axis, of which will be discussed below.

## Synthetic HDL products

As previously mentioned, the main focus of HDL research over the past several decades has been surrounding its role in cardiovascular disease. Because of its role in facilitating RCT, several “HDL mimetics” have been developed and tested clinically in humans for their ability to reduce the burden of atherosclerosis and number of events following an acute coronary event (66–69). These HDL-mimicking particles, termed reconstituted HDL (rHDL) or synthetic HDL (sHDL), are cholesterol-free HDL particles prepared from plasma purified or recombinantly expressed ApoA-I or short synthetic ApoA-I mimetic peptides complexed with phospholipids. Since they lack cholesterol, these “empty” particles are highly effective in effluxing cholesterol from lipid-laiden cells both *in vitro* and *in vivo* (9, 70, 71). In addition to their augmented efflux capacity, these particles offer a natural ability to target SR-BI-expressing cells. When combined, the ability of rHDL/sHDL to deplete cellular cholesterol, target SR-BI expressing cells, along with the biocompatibility of the individual components and proven clinical safety make the application of sHDL for cancer therapy increasingly attractive.

### Clinically tested sHDL products

The concept of utilizing sHDL and ApoA-I mimetic peptides as a cholesterol depletion therapy has been around for decades, but focused primarily in the context of cardiovascular diseases. In fact, several sHDL therapies have been developed and tested in various stages of human clinical trials (66, 69, 72). The purpose of such sHDL infusion therapies was to efflux cholesterol and reduce plaque size and vulnerability following an initial coronary event, in order to decrease the occurance of secondary events. Early sHDL clinical trials utilized lipid-free ApoA-I protein or mimetic peptides, such as ApoA-I milano, D-4F, and L-4F, however it was shown that the naked proteins and peptides themselves had a very short plasma half-life, and their pharmacological effect suffered as a consequence (73, 74). Formulation of peptide or full-length ApoA-I protein with phospholipid, forming sHDL, was shown to markedly improve plasma half-life and thus its overall therapeutic effect (75). Moreover, studies have shown that the phospholipid component of sHDL therapies is a driving determinant of the overall pharmacokinetic and pharmacodynamic effect (71).

Measurable improvements in the pharmacokinetic and pharmacodynamic effects of sHDL therapies has led to their progression from bench to bedside in both early and late stage clinical trials. Such sHDL products include peptide-based sHDLs, including ETC-642 (22A peptide/dipalmitoylphosphatidylcholine/sphingomyelin), and ApoA-I protein based sHDLs, including ETC-216 (recombinant ApoA-I/palmitoyloleoylphosphatidylcholine) and CER-001 (recombinant ApoA-I/sphingomyelin/dipalmitoylphosphatidylglycerol), among others. These products were all shown to be safe at high doses of up to 100 mg/kg in humans and possess potent cholesterol efflux abilities (76, 77). More recently CSL-112, reconstituted ApoA-I/soybean phosphatidylcholine, has advanced to a 17,000 patient Phase III clinical trial after showing promising ability to reduce atheroma burden and decrease secondary coronary events in earlier trials (78–80). Given their proven clinical safety and ability to facilitate cholesterol removal, sHDL products could be easily translated for use as cholesterol depleting therapies in cancer.

## sHDL for cancer therapy

### Cholesterol depletion

Given the dependence of endocrine cancers on cholesterol, cholesterol-targeting therapies have gained increasing attention. One approach is to directly deplete cholesterol from cells using cholesterol scavenging therapies. In addition to cholesterol being essential for the formation of new membranes during cell division, it is also vital for the formation of lipid-rafts in the plasma membrane. These lipid rafts are rich in cholesterol and sphingolipids and house many proteins and transporters involved in key signaling pathways, including the Akt signaling pathway implicated in the migration, proliferation, and survival of cancer cells (81). By depleting cholesterol from cells, lipid rafts are disrupted and the proteins they house internalized, drastically reducing the cell's ability to carry out its functions and often triggering cell death (4). In cancer cell lines, treatment with cyclodextrins induced marked cell death, and that cells with a higher abundance of lipid rafts were more susceptible to such treatments (82–84). More recently, Taylor et al. showed that HAC15 adrenal carcinoma cells treated with ETC-642, a clinically tested sHDL, displayed marked reduction in cellular cholesterol levels in addition to inhibition of aldosterone, cortisol, and androstenedione production (85). Thus, the application of cholesterol-depleting therapies, namely sHDL, for endocrine cancer deserves further investigation.

### sHDL peptides

Several recent studies have investigated the use of HDL-mimetics, including sHDL, ApoA-I protein, and ApoA-I mimetic peptides, for cancer treatment, outlined in Table 2. In addition, treatment of cells with both sHDL and chemotherapeutic drugs was able to reduce the overall effective dose (91). Other studies utilizing ApoA-I protein or mimetic peptides L-4F, L-5F, and D-4F have shown that treatment of tumor-bearing mice with either protein or peptide can reduce both tumor volume and angiogenesis in tumor tissues when compared to control mice (87–90, 92–94). For example, in a mouse model of ovarian cancer, Gao et. al. show that L-4F peptide, when administered subcutaneously at 10 mg/kg/day for 3 weeks, could reduce overall angiogenesis and vessel number within the tumor tissue, which was connected to a decrease in expression levels of hypoxia inducible factor-1α (HIF-1α) (88). In a similar study, they also show that L-5F peptide could exert similar anti-angiogensis effects and led to decreased levels of serum vascular endothelial growth factor (VEGF) (87). In a separate study utilizing a similar ovarian cancer mouse model, Su et. al. demonstrated that both L-4F and L-5F peptides could decrease the overall volume of tumors in both flank and intraperitoneal tumors when given 10 mg/kg/day over the course of 5 or 9 weeks, respectively (86). They postulate that the antitumorogenic effect could be related to peptides' abilities to reduce circulating levels of LPA, and they were found to have significantly greater binding affinity for LPA when compared to full length ApoA-I protein. In a study by Peng et. al. L-4F peptide given at 10 mg/kg/day for 1 week was able to not only reduce size and weight of H7 pancreatic tumors in mice, but also significantly reduce several markers of inflammation within the tissue as well (90).

**Table 2:** Experimental studies utilizing HDL for endocrine cancer therapy.

**Cancer**	**Treatment**	**Model**	**Major findings**	**References**
Ovarian	• L-4F peptide or sc-4F ctrl • [-] 10 mg/kg/day S.Q. for 5 wks, starting either the day of ID8 cell injection or 2 wks post-ID8 injection • L-5F peptide • [-] 10 mg/kg/day S.Q. for 5 wks (for S.Q. tumors) or 9 wks (for I.P. tumors) • Compared to buffer treated control mice • D-4F peptide • [-] 300 μg/mL (129.8 μM) in drinking water, starting immediately post-ID8 injection • [-] Compared to normal drinking water	• Female C57BL6/J mice, 9 wk old • ID8 cells given:•[-] S.Q. (5 wk flank tumor model) • [-] I.P. (9 wk tumor model)	• L-4F: • [-] Smaller flank tumor volumes when given both immediately following or 2 wks post-ID8 injection (5 wk S.Q. tumor model) • L-5F: • [-] Flank tumor size decreased (5 wk S.Q. tumor model) • [-] Number of tumor nodules decreased (9 wk I.P tumor model) • D-4F: • [-] Smaller flank tumor volume (5 wk S.Q. tumor model) • [-] Fewer number of tumor nodules (9 wk I.P. tumor model)	(86)
Ovarian	• L-5F peptide • [-] 10 mg/kg/day S.Q. for 5 wks, starting immediately after ID8 cell injections	• Female C57BL/6 mice • ID8 cells injected S.Q.	• Decreased the number of perfused vessels within tumors • Decreased size of total vessels within tumor • Decreased VEGF levels in both serum and tumor tissue	(87)
Ovarian	• L-4F peptide • [-] 10 mg/kg/day S.Q. for 3 wks, starting 2 weeks post-ID8 injection • [-] Compared to sc-4F peptide treated mice	• Female C57BL/6 mice, 9 wk old • ID8 cells injected S.Q.	• Decreased expression and activity of HIF-1α in tumors • Reduced the number of vessels and overall angiogenesis within tumors	(88)
Breast	• L-4F peptide • 10 mg/kg S.Q. daily from weaning to 45 days, and 3x per wk until 19 wks of age	• Mammary PyMT transgenic mice	• Significantly increased tumor latency and inhibited tumor development • Decreased plasma levels of oxLDL	(89)
Pancreatic	• L-4F peptide • [-] 10 mg/kg/day I.P. for 1 week, starting immediately post-H7 injection • [-] Compared to sc-4F peptide treated mice	• Female C57BL/6 mice, 6–8 w/o•H7 cells injected directly into pancreas	• Reduced tumor size and weight • Reduced number of inflammatory tumor infiltrating cells, including Th17 and Th1 lymphocytes • Decreased mRNA expression of inflammatory cytokines in tumors • Decreased % of M2 macrophage polarization in tumors	(90)

*D-4F, D-amino acid version of L-4F; I.P., intraperitoneal; L-4F, DWFKAFYDKVAEKFKEAF; L-5F, DWLKAFYDKVFEKFKEFF; oxLDL, oxidized low-density lipoprotein; PyMT, mammary tumor virus-polyoma middle T-antigen; sc-4F, scrambled-4F peptide; S.Q., subcutaneous; Th1, T-helper cell 1; Th17, T-helper cell 17; VEGF, vascular endothelial growth factor; wk, week*.

While the above studies also included extensive screening of ApoA-I and mimetic peptides for viability and anti-proliferative activity in a broad range of cancer cell lines *in vitro*, there are also reports describing the ability of HDL to induce proliferation, migration, and survival in cancer cell cultures (32, 33, 95, 96). Consistent with previously mentioned clinical findings showing a positive association between HDL-C and cancer risk, these studies support the notion that HDL-C may promote the progression of cancer by supplying the tumor cells with their increasing demand for cholesterol. However, a distinction should be made between the epidemiology of HDL and cancer and the utility of HDL in cancer treatment: namely, that the use of HDL in cancer therapy referred to in this review involves the administration of “empty” cholesterol-free particles. These particles are the nascent, discoidal HDL particles with high cholesterol efflux activity as proven both in basic and clinical research. Of course, studies utilizing plasma purified HDLs should be considered differently. Plasma HDLs contain a variety of different components, including signaling lipids responsible for many of HDL's pro-angiogenic and Akt-activating properties (97, 22), and namely cholesterol capable of being delivered to cells (98, 99). While such studies are integral to understanding the role of endogenous HDL in cancer pathogenesis, they should not be confused with therapeutic implications utilizing sHDL or mimetic peptides with a defined molecular makeup and superior cholesterol efflux capacity.

### Targeted drug delivery

Given the very poor solubility of many chemotherapeutic drugs, the hydrophobic lipid core of HDL presents an attractive environment and alternative strategy for delivery and formulation of this class of drugs. Not only is it possible to lower the overall dose of drug given by improving its solubility, but the SR-BI targeting ability of these sHDL nanoparticles affords the additional benefit of site-specific, cytosolic drug delivery to SR-BI over-expressing tumor cells while subsequently reducing systemic toxicity (94, 72, 100). Others have, with varying success, shown anti-tumorigenic by introducing HDL surface modifications to augment the targeting capacity and to extend particle half-life (101). The use of HDL-mimetics for targeted drug delivery has been extensively reviewed elsewhere (102, 72) and is beyond the scope of this review, however, its importance and growing relevance warrant mentioning.

## Summary and perspective

Decades of epidemiological evidence suggests that, notably -C, plays a role in the incidence and progression of cancer. Whether or not this role is causal or consequential, or whether the risk association is positive or negative under specific conditions is still left for debate. Despite, we know from years of clinical and basic cardiovascular research that is an intimate player in the RCT process and has specific and potent cholesterol efflux ability both *in vitro* and *in vivo*. We also know that cholesterol is a vital resource for cancer cells, which require a constant supply to maintain and facilitate their rapid proliferation and overall survival. Endocrine cancers, in particular, are at an increased demand for cholesterol given their additional need for steroid production making them even more susceptible to cholesterol depletion interventions and targeting by due to upregulation of SR-BI.

## Author contributions

All authors listed have made a substantial, direct and intellectual contribution to the work, and approved it for publication.

### Conflict of interest statement

The authors declare that the research was conducted in the absence of any commercial or financial relationships that could be construed as a potential conflict of interest.

## References

[1] Klimstra DS Modlin IR Coppola D Lloyd RV Suster S. The pathologic classification of neuroendocrine tumors: a review of nomenclature, grading, and staging systems. Pancreas (2010) 39:707–12. 10.1097/MPA.0b013e3181ec124e 20664470

[2] Hager MH Solomon KR Freeman MR. The role of cholesterol in prostate cancer. Curr Opin Clin Nutr Metab Care (2006) 9:379–85. 10.1097/01.mco.0000232896.66791.62 16778565

[3] Cruz PM Mo H McConathy WJ Sabnis N Lacko AG. The role of cholesterol metabolism and cholesterol transport in carcinogenesis: a review of scientific findings, relevant to future cancer therapeutics. Front Pharmacol. (2013). 4:119. 10.3389/fphar.2013.00119 24093019 PMC3782849

[4] Beloribi-Djefaflia S Vasseur S Guillaumond F. Lipid metabolic reprogramming in cancer cells. Oncogenesis 5:e189. 10.1038/oncsis.2015.49 26807644 PMC4728678

[5] Hu J Zhang Z Shen WJ Azhar S. Cellular cholesterol delivery, intracellular processing and utilization for biosynthesis of steroid hormones. Nutri Metab. (2010) 7:47. 10.1186/1743-7075-7-47 20515451 PMC2890697

[6] Pike LJ. Lipid rafts: bringing order to chaos. J Lipid Res. (2003) 44:655–67. 10.1194/jlr.R200021-JLR200 12562849

[7] Foster PA. Steroid metabolism in breast cancer. Minerva Endocrinol. (2008) 33:27–37. Available online at: http://cebp.aacrjournals.org/content/13/7/1185 18277377

[8] Purohit A Foster PA. Steroid sulfatase inhibitors for estrogen- and androgen-dependent cancers. J Endocrinol. (2012) 212:99–110. 10.1530/JOE-11-0266 21859802

[9] von Eckardstein A Nofer JR Assmann G High density lipoproteins and arteriosclerosis: role of cholesterol efflux and reverse cholesterol transport. Arterioscl Thromb Vasc Biol. (2001) 21:13–27. 10.1161/01.ATV.21.1.13 11145929

[10] Rye KA Barter PJ. Cardioprotective functions of HDLs. J Lipid Res. (2014) 55:168–79. 10.1194/jlr.R039297 23812558 PMC3886656

[11] Kontush A Lindahl M Lhomme M Calabresi L Chapman MJ Davidson WS Structure of HDL: particle subclasses and molecular components. In: von Eckardstein A Kardassis D editors. High Density Lipoproteins, vol. 224 Cham; Heidelberg; New York, NY; Dordrecht; London: Springer International Publishing (2015). p. 3–51. 10.1007/978-3-319-09665-0_1 25522985

[12] Lund-Katz S Phillips MC High density lipoprotein structure–function and role in reverse cholesterol transport. In: Harris JR editor. Cholesterol Binding and Cholesterol Transport Proteins, vol. 51 Netherlands: Springer (2010). p. 183–227. 10.1007/978-90-481-8622-8_7 PMC3215094 20213545

[13] Favari E Chroni A Tietge UJ Zanotti I Escolà-Gil JC Bernini F Cholesterol Efflux and Reverse Cholesterol Transport. In: von Eckardstein A Kardassis D editors. High Density Lipoproteins, vol. 224 Cham; Heidelberg; New York, NY; Dordrecht; London: Springer International Publishing (2015). p. 181–206. 10.1007/978-3-319-09665-0_4 25522988

[14] Zannis VI Fotakis P Koukos G Kardassis D Ehnholm C Jauhiainen M. HDL biogenesis, remodeling, and Catabolism. In: von Eckardstein A Kardassis D editors. High Density Lipoproteins, vol. 224. Cham; Heidelberg; New York, NY; Dordrecht; London: Springer International Publishing (2015). p. 53–111. 10.1007/978-3-319-09665-0_2 25522986

[15] Kontush A Chapman MJ High-Density Lipoproteins: Structure, Metabolism, Function and Therapeutics. Hoboken, NJ: John Wiley & Sons (2011).

[16] Kratzer A Giral H Landmesser U. High-density lipoproteins as modulators of endothelial cell functions: alterations in patients with coronary artery disease. Cardiovasc Res. (2014) 103:350–61. 10.1093/cvr/cvu139 24935432

[17] Heinecke JW. The HDL proteome: a marker–and perhaps mediator–of coronary artery disease. J Lipid Res. (2009) 50(Suppl.):S167–71. 10.1194/jlr.R800097-JLR200 19060251 PMC2674735

[18] Alwaili K Bailey D Awan Z Bailey SD Ruel I Hafiane A. The HDL proteome in acute coronary syndromes shifts to an inflammatory profile. Biochim Biophys Acta (2012) 1821:405–15. 10.1016/j.bbalip.2011.07.013 21840418

[19] Van Lenten BJ Hama SY de Beer FC Stafforini DM McIntyre TM Prescott SM Anti-inflammatory HDL becomes pro-inflammatory during the acute phase response. Loss of protective effect of HDL against LDL oxidation in aortic wall cell cocultures. J Clin Invest. (1995) 96:2758–67. 10.1172/JCI118345 8675645 PMC185985

[20] Shah AS Tan L Long JL Davidson WS. Proteomic diversity of high density lipoproteins: our emerging understanding of its importance in lipid transport and beyond. J Lipid Res. (2013) 54:2575–85. 10.1194/jlr.R035725 23434634 PMC3770071

[21] Birner-Gruenberger R Schittmayer M Holzer M Marsche G. Understanding high-density lipoprotein function in disease: recent advances in proteomics unravel the complexity of its composition and biology. Prog Lipid Res. (2014) 56:36–46. 10.1016/j.plipres.2014.07.003 25107698

[22] Kontush A Lhomme M Chapman MJ. Unraveling the complexities of the HDL lipidome. J Lipid Res. (2013) 54:2950–63. 10.1194/jlr.R036095 23543772 PMC3793600

[23] Baker DL Morrison P Miller B Riely CA Tolley B Westermann AM. Plasma lysophosphatidic acid concentration and ovarian cancer. JAMA (2002) 287:3081–2. 10.1001/jama.287.23.3081 12069669

[24] Sutphen R Xu Y Wilbanks GD Fiorica J Grendys EC LaPolla JP. Lysophospholipids are potential biomarkers of ovarian cancer. Cancer Epidemiol Biomarkers Prev. (2004) 13:1185–91. 15247129

[25] Willier S Butt E Grunewald TG. Lysophosphatidic acid (LPA) signalling in cell migration and cancer invasion: a focussed review and analysis of LPA receptor gene expression on the basis of more than 1700 cancer microarrays. Biol Cell (2013) 105:317–33. 10.1111/boc.201300011 23611148

[26] McMahon M Grossman J FitzGerald J Dahlin-Lee E Wallace DJ Thong BY. Proinflammatory high-density lipoprotein as a biomarker for atherosclerosis in patients with systemic lupus erythematosus and rheumatoid arthritis. Arthritis Rheum. (2006) 54:2541–9. 10.1002/art.21976 16868975

[27] Navab M Anantharamaiah GM Reddy ST Van Lenten BJ Fogelman AM. HDL as a biomarker, potential therapeutic target, and therapy. Diabetes (2009) 58:2711–7. 10.2337/db09-0538 19940234 PMC2780869

[28] Namiri-Kalantari R Gao F Chattopadhyay A Wheeler AA Navab KD Farias-Eisner R. The dual nature of HDL: anti-inflammatory and pro-inflammatory. Biofactors (2015) 41:153–9. 10.1002/biof.1205 26072738

[29] Rosenson RS Brewer HB Jr Ansell BJ Barter P Chapman MJ Heinecke JW. Dysfunctional HDL and atherosclerotic cardiovascular disease. Nat Rev Cardiol. (2015) 13:48–60. 10.1038/nrcardio.2015.124 26323267 PMC6245940

[30] Vaisar T Tang C Babenko I Hutchins P Wimberger J Suffredini AF. Inflammatory remodeling of the HDL proteome impairs cholesterol efflux capacity. J Lipid Res. (2015) 56:1519–30. 10.1194/jlr.M059089 25995210 PMC4513993

[31] Smith CK Seto NL Vivekanandan-Giri A Yuan W Playford MP Manna Z. Lupus high-density lipoprotein induces proinflammatory responses in macrophages by binding lectin-like oxidised low-density lipoprotein receptor 1 and failing to promote activating transcription factor 3 activity. Ann Rheum Dis. (2016). 76:602–11. 10.1136/annrheumdis-2016-209683 27543414 PMC6109980

[32] Pan B Ren H He Y Lv X Ma Y Li J. HDL of patients with type 2 diabetes mellitus elevates the capability of promoting breast cancer metastasis. Clin Cancer Res. (2012) 18:1246–56. 10.1158/1078-0432.CCR-11-0817 22261802

[33] Pan B Ren H Lv X Zhao Y Yu B He Y. Hypochlorite-induced oxidative stress elevates the capability of HDL in promoting breast cancer metastasis. J Transl Med. (2012) 10:65. 10.1186/1479-5876-10-65 22462581 PMC3342142

[34] Lane DM Boatman KK McConathy WJ. Serum lipids and apolipoproteins in women with breast masses. Breast Cancer Res Treat. (1995) 34:161–9. 10.1007/BF00665788 7647333

[35] Moorman PG Hulka BS Hiatt RA Krieger N Newman B Vogelman JH. Association between high-density lipoprotein cholesterol and breast cancer varies by menopausal status. Cancer Epidemiol Biomark Prev. (1998) 7:483–8. 9641492

[36] Fiorenza AM Branchi A Sommariva D. Serum lipoprotein profile in patients with cancer. A comparison with non-cancer subjects. Int J Clin Lab Res. (2000) 30:141–5. 10.1007/s005990070013 11196072

[37] Furberg AS Veierød MB Wilsgaard T Bernstein L Thune I. Serum high-density lipoprotein cholesterol, metabolic profile, and breast cancer risk. JNCI (2004) 96:1152–60. 10.1093/jnci/djh216 15292387

[38] Chang SJ Hou MF Tsai SM Wu SH Hou LA Ma H. The association between lipid profiles and breast cancer among Taiwanese women. Clin Chem Lab Med. 45:1219–23. 10.1515/CCLM.2007.263 17663634

[39] Cust AE Kaaks R Friedenreich C Bonnet F Laville M Tjønneland A. Metabolic syndrome, plasma lipid, lipoprotein and glucose levels, and endometrial cancer risk in the European Prospective Investigation into Cancer and Nutrition (EPIC). Endocrine Relat Cancer (2007) 14:755–67. 10.1677/ERC-07-0132 17914105

[40] Ahn J Lim U Weinstein SJ Schatzkin A Hayes RB Virtamo J. Prediagnostic total and high-density lipoprotein cholesterol and risk of cancer. Cancer Epidemiol Biomark Prev. (2009) 18:2814–21. 10.1158/1055-9965.EPI-08-1248 19887581 PMC3534759

[41] Jafri H Alsheikh-Ali AA Karas RH. Baseline and on-treatment high-density lipoprotein cholesterol and the risk of cancer in randomized controlled trials of lipid-altering therapy. J Am College Cardiol. (2010) 55:2846–54. 10.1016/j.jacc.2009.12.069 20579542

[42] Kitahara CM Berrington de González A Freedman ND Huxley R Mok Y Jee SH. Total cholesterol and cancer risk in a large prospective study in Korea. J Clin Oncol. (2011) 29:1592–8. 10.1200/JCO.2010.31.5200 21422422 PMC3082977

[43] Van Hemelrijck M Walldius G Jungner I Hammar N Garmo H Binda E Low levels of apolipoprotein A-I and HDL are associated with risk of prostate cancer in the Swedish AMORIS study. Cancer Causes Control (2011) 22:1011–9. 10.1007/s10552-011-9774-z 21562751

[44] Zhao W Guan J Horswell R Li W Wang Y Wu X. HDL cholesterol and cancer risk among patients with type 2 diabetes. Diabetes Care (2014) 37:3196–203. 10.2337/dc14-0523 25216507 PMC4237978

[45] Touvier M Fassier P His M Norat T Chan DS Blacher J. Cholesterol and breast cancer risk: a systematic review and meta-analysis of prospective studies. Br J Nutr. (2015) 114:347–57. 10.1017/S000711451500183X 26173770

[46] Chandler PD Song Y Lin J Zhang S Sesso HD Mora S. Lipid biomarkers and long-term risk of cancer in the Women's Health Study. Am J Clin Nutr. (2016) 103:1397–407. 10.3945/ajcn.115.124321 27099252 PMC4880994

[47] Guo S He X Chen Q Yang G Yao K Dong P. The effect of preoperative apolipoprotein A-I on the prognosis of surgical renal cell carcinoma: a retrospective large sample study. Medicine (2016) 95:e3147. 10.1097/MD.0000000000003147 27015197 PMC4998392

[48] Katzke VA Sookthai D Johnson T Kühn T Kaaks R. Blood lipids and lipoproteins in relation to incidence and mortality risks for CVD and cancer in the prospective EPIC–Heidelberg cohort. BMC Med. (2017) 15:218. 10.1186/s12916-017-0976-4 29254484 PMC5735858

[49] Li X Tang H Wang J Xie X Liu P Kong Y. The effect of preoperative serum triglycerides and high-density lipoprotein-cholesterol levels on the prognosis of breast cancer. Breast (2017) 32:1–6. 10.1016/j.breast.2016.11.024 27939967

[50] Zhou P Li B Liu B Chen T Xiao J. Prognostic role of serum total cholesterol and high-density lipoprotein cholesterol in cancer survivors: a systematic review and meta-analysis. Clin Chim Acta (2018) 477:94–104. 10.1016/j.cca.2017.11.039 29223765

[51] Kucharska-Newton AM Rosamond WD Mink PJ Alberg AJ Shahar E Folsom AR. HDL-cholesterol and incidence of breast cancer in the ARIC cohort study. Ann Epidemiol. (2008) 18:671–7. 10.1016/j.annepidem.2008.06.006 18794007 PMC2566531

[52] Ehmann M Felix K Hartmann D Schnölzer M Nees M Vorderwülbecke S. Identification of potential markers for the detection of pancreatic cancer through comparative serum protein expression profiling. Pancreas (2007) 34:205–14. 10.1097/01.mpa.0000250128.57026.b2 17312459

[53] Zhang Z Bast RC Yu Y Li J Sokoll LJ Rai AJ. Three biomarkers identified from serum proteomic analysis for the detection of early stage ovarian cancer. Cancer Res. (2004) 64:5882–90. 10.1158/0008-5472.CAN-04-0746 15313933

[54] Kozak KR Amneus MW Pusey SM Su F Luong MN Luong SA. Identification of biomarkers for ovarian cancer using strong anion-exchange ProteinChips: potential use in diagnosis and prognosis. Proc Natl Acad Sci USA. (2003) 100:12343–8. 10.1073/pnas.2033602100 14523236 PMC218760

[55] Kozak KR Su F Whitelegge JP Faull K Reddy S Farias-Eisner R. Characterization of serum biomarkers for detection of early stage ovarian cancer. Proteomics (2005) 5:4589–96. 10.1002/pmic.200500093 16237736

[56] Moore LE Fung ET McGuire M Rabkin CC Molinaro A Wang Z. Evaluation of apolipoprotein A1 and posttranslationally modified forms of transthyretin as biomarkers for ovarian cancer detection in an independent study population. Cancer Epidemiol Biomark Prev. (2006) 15:1641–6. 10.1158/1055-9965.EPI-05-0980 16985025

[57] Clarke CH Yip C Badgwell D Fung ET Coombes KR Zhang Z. Proteomic biomarkers apolipoprotein A1, truncated transthyretin and connective tissue activating protein III enhance the sensitivity of CA125 for detecting early stage epithelial ovarian cancer. Gynecol Oncol. (2011) 122:548–53. 10.1016/j.ygyno.2011.06.002 21708402 PMC3152646

[58] Kim YW Bae SM Lim H Kim YJ Ahn WS. Development of multiplexed bead-based immunoassays for the detection of early stage ovarian cancer using a combination of serum biomarkers. PLoS ONE (2012) 7:e44960. 10.1371/journal.pone.0044960 22970327 PMC3438175

[59] Tuft Stavnes H Nymoen DA Hetland Falkenthal TE Kærn J Tropé CG Davidson B (APOA1 mRNA expression in ovarian serous carcinoma effusions is a marker of longer survival. Am J Clin Pathol. 2014) 142:51–7. 10.1309/AJCPD8NBSHXRXQL7 24926085

[60] Martin LJ Melnichouk O Huszti E Connelly PW Greenberg CV Minkin S Serum lipids, lipoproteins, and risk of breast cancer: a nested case-control study using multiple time points. JNCI J Natl Cancer Institute (2015) 107:djv032. 10.1093/jnci/djv032 PMC4822522 25817193

[61] Hafiane A Genest J. High density lipoproteins: measurement techniques and potential biomarkers of cardiovascular risk. BBA Clin. (2015) 3:175–88. 10.1016/j.bbacli.2015.01.005 26674734 PMC4661556

[62] Gutierrez-Pajares JL Ben Hassen C Chevalier S Frank PG. SR-BI: linking cholesterol and lipoprotein metabolism with breast and prostate cancer. Front Pharmacol. (2016) 7:338. 10.3389/fphar.2016.00338 27774064 PMC5054001

[63] Li J Wang J Li M Yin L Li XA Zhang TG. Up-regulated expression of scavenger receptor class B type 1 (SR-B1) is associated with malignant behaviors and poor prognosis of breast cancer. Pathol Res Pract. (2016) 212:555–9. 10.1016/j.prp.2016.03.011 27067809

[64] Yuan B Wu C Wang X Wang D Liu H Guo L. High scavenger receptor class B type I expression is related to tumor aggressiveness and poor prognosis in breast cancer. Tumour Biol. (2016) 37:3581–8. 10.1007/s13277-015-4141-4 26456958

[65] Ruscica M Botta M Ferri N Giorgio E Macchi C Franceschini G. High density lipoproteins inhibit oxidative stress-induced prostate cancer cell proliferation. Sci Rep. (2018) 8:2236. 10.1038/s41598-018-19568-8 29396407 PMC5797231

[66] Nissen SE Tsunoda T Tuzcu EM Schoenhagen P Cooper CJ Yasin M. Effect of recombinant apoa-i milano on coronary atherosclerosis in patients with acute coronary syndromes: a randomized controlled trial. JAMA (2003) 290:2292–300. 10.1001/jama.290.17.2292 14600188

[67] Remaley AT Amar M Sviridov D. HDL-replacement therapy: mechanism of action, types of agents and potential clinical indications. Exp Rev Cardiovasc Ther. (2008) 6:1203–15. 10.1586/14779072.6.9.1203 18939908 PMC4164165

[68] Dasseux JL Schwendeman AS Zhu L Apolipoprotein A-I Mimics. USPTO. United States, Cerenic Therapeutics Holding SA (2013).

[69] Krause BR Remaley AT. Reconstituted HDL for the acute treatment of acute coronary syndrome. Curr Opin Lipidol. (2013) 24:480–6. 10.1097/MOL.0000000000000020 24184938

[70] Di Bartolo BA Nicholls SJ Bao S Rye KA Heather AK Barter PJ The apolipoprotein A-I mimetic peptide ETC-642 exhibits anti-inflammatory properties that are comparable to high density lipoproteins. Atherosclerosis (2011) 217:395–400. 10.1016/j.atherosclerosis.2011.04.001 21571275

[71] Schwendeman A Sviridov DO Yuan W Guo Y Morin EE Yuan Y. The effect of phospholipid composition of reconstituted HDL on its cholesterol efflux and anti-inflammatory properties. J Lipid Res. (2015) 56:1727–37. 10.1194/jlr.M060285 26117661 PMC4548777

[72] Kuai R Li D Chen YE Moon JJ Schwendeman A. High-density lipoproteins: nature's multifunctional nanoparticles. ACS Nano (2016) 10:3015–41. 10.1021/acsnano.5b07522 26889958 PMC4918468

[73] Nanjee MN Crouse JR King JM Hovorka R Rees SE Carson ER. Effects of intravenous infusion of lipid-free apo A-I in humans. Arterioscler Thromb Vasc Biol. (1996) 16:1203–14. 10.1161/01.ATV.16.9.1203 8792776

[74] Li D Gordon S Schwendeman A Remaley A Apolipoprotein mimetic peptides for stimulating cholesterol efflux. In: Anantharamaiah GM Goldberg D editors. Apolipoprotein Mimetics in the Management of Human Disease. Cham; Heidelberg; New York, NY; Dordrecht; London: Springer International Publishing (2015). p. 29–42.

[75] Tang J Li D Drake L Yuan W Deschaine S Morin EE. Influence of route of administration and lipidation of apolipoprotein A-I peptide on pharmacokinetics and cholesterol mobilization. J Lipid Res. (2017) 58:124–36. 10.1194/jlr.M071043 27881716 PMC5234715

[76] Khan MLN Drake SL Crockatt JG Dasseux JLH Single-dose intravenous infusion of ETC-642, a 22-Mer ApoA-I analogue and phospholipids complex, elevates HDL-C in atherosclerosis patients. Circulation (2003) 108(Suppl. IV):563–4.

[77] Miles J Khan M Painchaud C Lalwani N Drake S Dasseux J Single-dose tolerability, pharmacokinetics, and cholesterol mobilization in HDL-C fraction following intravenous administration of ETC-642, a 22-mer ApoA-I analogue and phospholipids complex, in atherosclerosis patients. Arterioscler Thromb Vasc Biol. (2004), E19–E19.

[78] CSL (2018). CSL Behring Announces First Patient Enrollment in Phase 3 Clinical Trial of CSL112 to Assess Reduction of Early Recurrent Cardiovascular Events in Heart Attack Survivors. King of Prussia, PA: PR Newswire.

[79] Easton R Gille A D'Andrea D Davis R Wright SD Shear C. A multiple ascending dose study of CSL112, an infused formulation of ApoA-I. J Clin Pharmacol. (2014) 54:301–10. 10.1002/jcph.194 24122814

[80] Gille A D'Andrea D Tortorici MA Hartel G Wright SD. CSL112 (apolipoprotein A-I [human]) enhances cholesterol efflux similarly in healthy individuals and stable atherosclerotic disease patients. Arterioscler Thromb Vasc Biol. (2018) 38:953–63. 10.1161/ATVBAHA.118.310538 29437574 PMC5895137

[81] Badana A Chintala M Varikuti G Pudi N Kumari S Kappala VR. Lipid raft integrity is required for survival of triple negative breast cancer cells. J Breast Cancer (2016) 19:372–84. 10.4048/jbc.2016.19.4.372 28053625 PMC5204043

[82] Li YC Park MJ Ye SK Kim CW Kim YN. Elevated levels of cholesterol-rich lipid rafts in cancer cells are correlated with apoptosis sensitivity induced by cholesterol-depleting agents. Am J Pathol. (2006) 168:1107–18; quiz 1404–1105. 10.2353/ajpath.2006.050959 16565487 PMC1606567

[83] Resnik N Repnik U Kreft ME Sepčić K Maček P Turk B. Highly selective anti-cancer activity of cholesterol-interacting agents methyl-beta-cyclodextrin and ostreolysin A/pleurotolysin b protein complex on urothelial cancer cells. PLoS ONE (2015) 10:e0137878. 10.1371/journal.pone.0137878 26361392 PMC4567298

[84] Yamaguchi R Perkins G Hirota K. Targeting cholesterol with beta-cyclodextrin sensitizes cancer cells for apoptosis. FEBS Lett. (2015) 589 (24 Pt B):4097–105. 10.1016/j.febslet.2015.11.009 26606906

[85] Taylor MJ Sanjanwala AR Morin EE Rowland-Fisher E Anderson K Schwendeman A. Synthetic high-density lipoprotein (sHDL) inhibits steroid production in HAC15 adrenal cells. Endocrinology (2016) 157:3122–9. 10.1210/en.2014-1663 27253994 PMC4967112

[86] Su F Kozak KR Imaizumi S Gao F Amneus MW Grijalva V. Apolipoprotein A-I (apoA-I) and apoA-I mimetic peptides inhibit tumor development in a mouse model of ovarian cancer. Proc Natl Acad Sci USA. (2010) 107:19997–20002. 10.1073/pnas.1009010107 21041624 PMC2993420

[87] Gao F Vasquez SX Su F Roberts S Shah N Grijalva V. L-5F, an apolipoprotein A-I mimetic, inhibits tumor angiogenesis by suppressing VEGF/basic FGF signaling pathways(). Integr Biol. (2011) 3:479–89. 10.1039/c0ib00147c 21283904 PMC3248743

[88] Gao F Chattopadhyay A Navab M Grijalva V Su F Fogelman AM. Apolipoprotein A-I mimetic peptides inhibit expression and activity of hypoxia-inducible factor-1α in human ovarian cancer cell lines and a mouse ovarian cancer model. J Pharmacol Exp Ther. (2012) 342:255–62. 10.1124/jpet.112.191544 22537771 PMC3400800

[89] Cedó L García-León A Baila-Rueda L Santos D Grijalva V Martínez-Cignoni MR. ApoA-I mimetic administration, but not increased apoA-I-containing HDL, inhibits tumour growth in a mouse model of inherited breast cancer. Sci Rep. (2016) 6:36387. 10.1038/srep36387 27808249 PMC5093413

[90] Peng M Zhang Q Cheng Y Fu S Yang H Guo X. Apolipoprotein A-I mimetic peptide 4F suppresses tumor-associated macrophages and pancreatic cancer progression. Oncotarget (2017) 8:99693–706. 10.18632/oncotarget.21157 29245934 PMC5725125

[91] Subramanian C Kuai R Zhu Q White P Moon J Schwendeman A sHDL nanoparticles: a novel therapeutic strategy for adrenocortical carcinomas. Surgery (2016) 159:284–95. 10.1016/j.surg.2015.08.023 26582501 PMC4709032

[92] Su F Grijalva V Navab K Ganapathy E Meriwether D Imaizumi S. HDL mimetics inhibit tumor development in both induced and spontaneous mouse models of colon cancer. Mol Cancer Ther. (2012) 11:1311–9. 10.1158/1535-7163.MCT-11-0905 22416044 PMC3374063

[93] Zamanian-Daryoush M Lindner D Tallant TC Wang Z Buffa J Klipfell E. The cardioprotective protein apolipoprotein a1 promotes potent anti-tumorigenic effects. J Biol Chem. (2013) 288:21237–52. 10.1074/jbc.M113.468967 23720750 PMC3774392

[94] Zheng Y Liu Y Jin H Pan S Qian Y Huang C. Scavenger receptor B1 is a potential biomarker of human nasopharyngeal carcinoma and its growth is inhibited by HDL-mimetic nanoparticles. Theranostics (2013) 3:477–86. 10.7150/thno.6617 23843895 PMC3706691

[95] Sekine Y Demosky SJ Stonik JA Furuya Y Koike H Suzuki K. High-density lipoprotein induces proliferation and migration of human prostate androgen-independent cancer cells by an ABCA1-dependent mechanism. Mol Cancer Res. (2010) 8:1284–94. 10.1158/1541-7786.MCR-10-0008 20671065 PMC2941551

[96] Danilo C Gutierrez-Pajares JL Mainieri MA Mercier I Lisanti MP Frank PG. Scavenger receptor class B type I regulates cellular cholesterol metabolism and cell signaling associated with breast cancer development. Breast Cancer Res. (2013) 15:R87. 10.1186/bcr3483 24060386 PMC3978612

[97] Nofer JR Kehrel B Fobker M Levkau B Assmann G von Eckardstein A. HDL and arteriosclerosis: beyond reverse cholesterol transport. Atherosclerosis (2002) 161:1–16. 10.1016/S0021-9150(01)00651-7 11882312

[98] Pussinen PJ Karten B Wintersperger A Reicher H McLean M Malle E. The human breast carcinoma cell line HBL-100 acquires exogenous cholesterol from high-density lipoprotein via CLA-1 (CD-36 and LIMPII analogous 1)-mediated selective cholesteryl ester uptake. Biochem J. (2000) 349 (Pt 2):559–66. 10.1042/bj3490559 10880355 PMC1221179

[99] Connelly MA Williams DL. SR-BI and cholesterol uptake into steroidogenic cells. Trends Endocrinol Metab. (2003) 14:467–72. 10.1016/j.tem.2003.10.002 14643062

[100] Mooberry LK Sabnis NA Panchoo M Nagarajan B Lacko AG. Targeting the SR-B1 receptor as a gateway for cancer therapy and imaging. Front Pharmacol. (2016) 7:466. 10.3389/fphar.2016.00466 28018216 PMC5156841

[101] Tang J Kuai R Yuan W Drake L Moon JJ Schwendeman A. Effect of size and pegylation of liposomes and peptide-based synthetic lipoproteins on tumor targeting. Nanomedicine (2017) 13:1869–78. 10.1016/j.nano.2017.04.009 28434931 PMC5554706

[102] Foit L Giles FJ Gordon LI Thaxton CS. Synthetic high-density lipoprotein-like nanoparticles for cancer therapy. Expert Rev Anticancer Ther. (2015) 15:27–34. 10.1586/14737140.2015.990889 25487833 PMC4638421

